# Investigating the dimensions of globalization and its impact on poverty in Iran: An improved bat algorithm approach

**DOI:** 10.1016/j.mex.2021.101210

**Published:** 2021-01-05

**Authors:** Fatima Nazari Robati, Hossein Akbarifard, Seyyed abdolmajid Jalaee

**Affiliations:** Department of Economics, Faculty of Management and Economics, Shahid Bahonar University of Kerman, Kerman, Iran

**Keywords:** Globalization, Poverty, Improved bat algorithm, Optimization

## Abstract

Collective intelligence is one of the most powerful optimization techniques based on group behaviors of organisms. Bat algorithm (BA) is an algorithm inspired by the collective action of bats in the wild, presented in 2010 by Yang. Researchers have made several efforts to improve these algorithms. This article investigates the effect of globalization on Iran's poverty by enhancing the performance of BA. As an inescapable reality, globalization has various political, social, and economic dimensions, each with different effects on poverty. In this article, to improve the algorithm's performance, the speed and motion relationships of bats were modified such that to adapt the movement of bats as optimization solutions toward the target. The mutation operator is also used to check all points of the search space to get rid of the optimal local optimization. The study period is the years 1995 to 2017. The results showed that globalization affects Iran's poverty in various dimensions, and the performance of the improved bat collective intelligence algorithm (ISABA) for modeling is better than that of the bat algorithms (BAs).

• This article provides a suitable method for researchers to study poverty.
• Improved BAs can help researchers solve complex problems.
• The results obtained by the collective intelligence algorithms in this paper help researchers in the field of poverty to compare the results of their research with it.

## Graphical abstract

**Figure:**
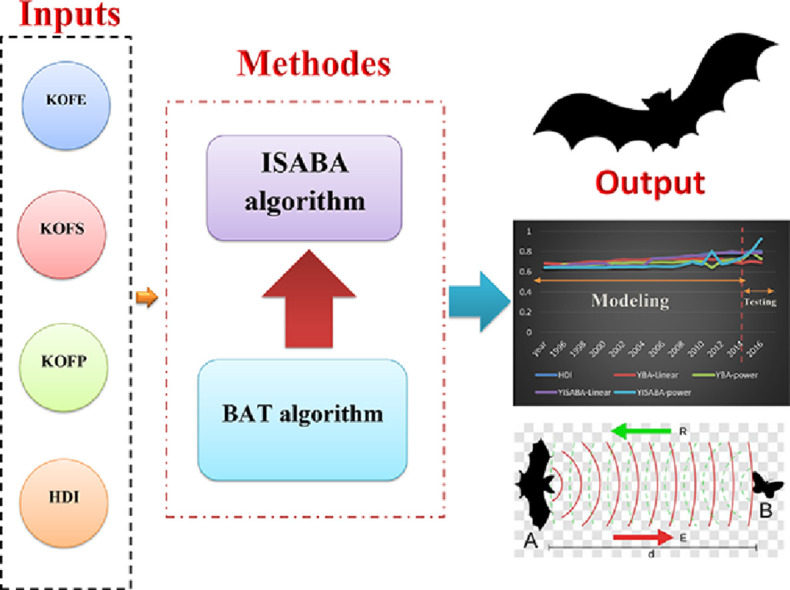

Specifications table

**Table:** 

Subject Area	Economics and Finance
More specific subject area	*Macroeconomics - Monetary Economics*
Method name	*Improved bat collective intelligence algorithm (ISABA)*
Name and reference of the original method	***BA****:* Yang, X.S. *A new Metaheuristic Bat-Inspired Algorithm, in: Nature Inspired Cooperative Strategies for Optimization Studies in Computational Intelligence Vol*. 284, Springer Berlin, 2010, pp. 65-74.
Resource availability	**:**https://info.worldbank.org/governance/wgi/ https://www.heritage.org/index/explore?view=by-region-country-year&u=637188530188497349

## Introduction

Globalization, as one of the most critical issues in the present era, is the interrelationship between governments and societies that has led to the creation of the current world order. This is indeed a process by which events, decisions, and activities in any part of the world can have significant consequences for other individuals and communities in other parts of the world [1]. Globalization has economic, social, and political dimensions. Liberalization and globalization increase the volume and variety of exchanges of goods and services, enhance the flow of international capital, and accelerate the transfer of technology [2]. One of the controversial issues in the globalization literature is its effect on the living conditions of income groups or, more precisely, on poverty and income distribution among income groups. Some scholars believe that there is a positive relationship between poverty reduction and trade liberalization [3,4]. Meanwhile, some others, such as Bhasin, find a negative correlation in this regard. But, many economists believe that open economies perform better in the long run than closed economies [5]. However, it is still feared that trade liberalization will further hurt the weaker groups in the marketplace and despite the probable success of open regimes, some people remain poor. Accordingly, the purpose of this article is to investigate the impact of different dimensions of globalization on poverty in Iran. This discussion is very complicated. So far, various studies have been conducted in this field. In this article, an attempt is made to provide a suitable model for poverty in Iran by improving the bat collective intelligence algorithm. Many nature-inspired algorithms have been developed to solve severe problems in optimization problems. In general, two examples of natural behaviors have been used to create algorithms. Evolutionary algorithms and algorithms are based on collective intelligence [6]. Collective intelligence, in turn, is a decentralized and self-organized behavior that occurs naturally or artificially among beings. BA algorithms are among the cooperative intelligence algorithms based on microbats hunting behavior that was introduced by Yang in 2010 [7].

BA uses the frequency propagation rate of the bats to find the answer. As a result, this algorithm is efficient because of its fast start. Standard BA algorithms operate based on the orientation feature of bats. There are many areas for improving the performance of this algorithm. To improve performance, researchers have used a variety of methods. Kumaraswamy and Wahi proposed a combination of BA algorithms and the K-means algorithm (KMBA) for better clustering [8]. Lin et al. proposed chaotic BA algorithms for estimating parameters in dynamic biological systems [9]. Nakamura et al. developed discrete algorithms to solve clustering and feature selection problems [10]. Jamil et al. improved the BA algorithms by varying the altitude and propagation rate and combining them with Levy flight. They determined the efficiency of the algorithm by experimenting with various functions [11]. Zhang and Wang used mutation to diversify the solution search space and used this algorithm to process the image [12]. Wang solved numerical problems and benchmark combinations by combining BA algorithms and harmonic search [13]. Fister et al. provided a differential evolution for this purpose [14]. Fister et al. proposed four algorithms (QBA) for geometric calculations and large-scale optimization problems [15].

According to the mentioned points, in this article, by improving the BA's performance, a suitable model is presented for poverty in Iran, in which the role of globalization is prominent. The remainder of this paper is organized as follows: After providing a brief introduction, BA algorithms are introduced. Improvements made in the structure of BA algorithms are then added. Finally, the output of research models is examined based on statistical scales.

### *Method details

**BA** [7]

**Fig. 1:**
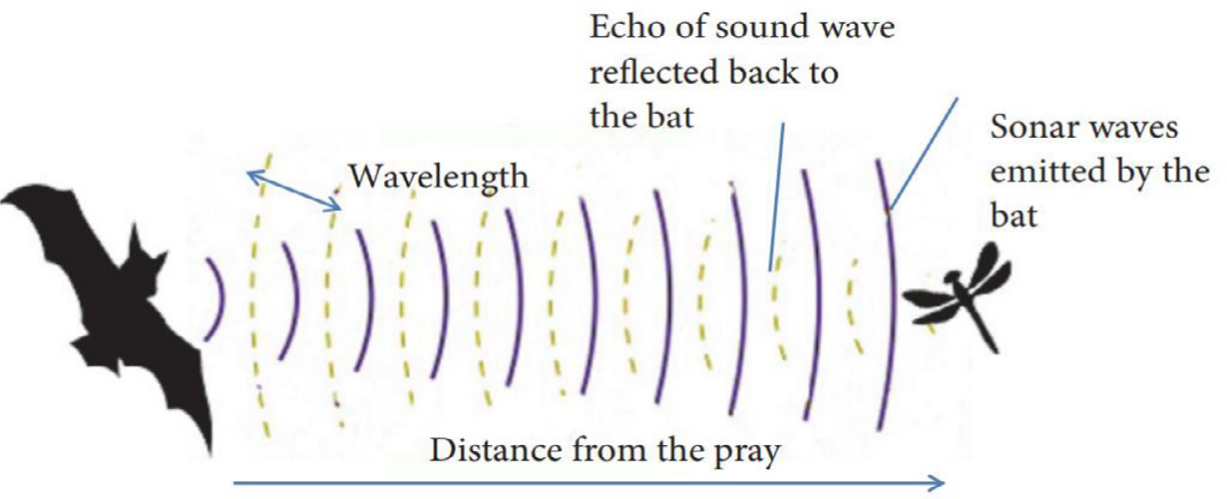
Echolocation behavior of the microbats. Fig. notes: This Fig. shows how to receive sonar waves reflected from prey to microbats. Each micro-bat can identify its food by sending sonar waves to its surroundings and re-reading the reflection of these waves from the environment.

BA algorithms were introduced by Young. This algorithm employs the echo detection property of microbats. Microbats create a three-dimensional image of their surroundings by emitting sound pulses at a certain speed and frequency and receiving the reflected pulse, which is possible by delaying the pulse. Each bat can send about 10 to 20 pulses at a specific rate per second. In this way, bats identify the exact location of their prey [16]. Fig. 1 shows the performance of microbats.

These bats use wavelengths between 0.7 and 17 mm and a variable frequency between 25 and 150 kHz. Bats can change the pulse emission rate from 0 to 1 [17,^18^,19].

Given the peculiarity of bats, Young established BAs based on the following three constitutions [19]:

• All bats use orientation to detect the environment and understand the difference between prey and other obstacles using voice tracking.
• Bats are randomly searching for prey at speed *vi* , position *xi*, and frequency *fmin*, with wavelength λ and loans $A_{0}$. They can also automatically adjust the wavelength of the emitted pulses and their pulse emission rate matches according to the proximity of their prey.
• However, the volume can be changed in different ways; for example, from $A_{0}\;$to $A_{min}$.

## The movement of bats

Each bat moves at the speed of $v_{i}^{t}$ and position of $x_{i}^{t}$. It repeats this pattern in the next d search space to find its prey. Of all the bats, there is only one optimal solution $x_{*}$. These three rules can be expressed in Eqs. (1–3).

(1) $$f_{i}=f_{min}+\left(f_{max}-f_{min}\right)\beta$$

(2) $$v_{i}^{t}=v_{i}^{t-1}+\left(x_{i}^{t-1}-x_{*}\right)f_{i}$$

(3) $$x_{i}^{t}=x_{i}^{t-1}+v_{i}^{t}$$

where β∈ [0 ~ 1] is a random vector obtained from a uniform distribution.

## $A_{i}$volume and $r_{i}$sound emission rate

To improve the performance of the BA, the propagation rate in the iterations can be changed. Because each bat reduces the volume and increases the pulse emission rate by finding its prey, the noise values and pulse emission rate for the bats change in Eqs. (4–6).

(4) $$A_{i}^{t+1}=\propto A_{i}^{t}$$

(5) $$r_{i}^{t+1}=r_{i}^{0}\left[1-\text{exp}\left(-\gamma t\right)\right]$$

(6) $$A_{i}^{t}\to 0\;r_{i}^{t}\to r_{i}^{0}\;as\;t\to \infty$$

where α and γ coefficients are constant, 0 < *α* < 1, and γ > 0; however, according to experiments, α values were considered to be between 0.9 and 0.98.

**Fig. 2:**
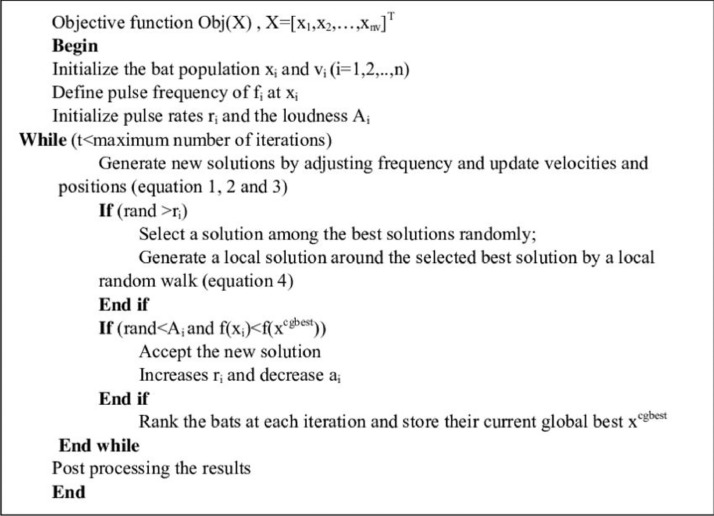
Pseudo-code of Bat Algorithm. Fig. notes: In this pseudo-code, each of the steps of the Bat algorithm is introduced and all the steps of executing a loop of the algorithm are explained.

Based on the above rules and the high relations, the operation steps of BA algorithms are expressed in the form of pseudo-code in Fig. 2.

**Fig. 3:**
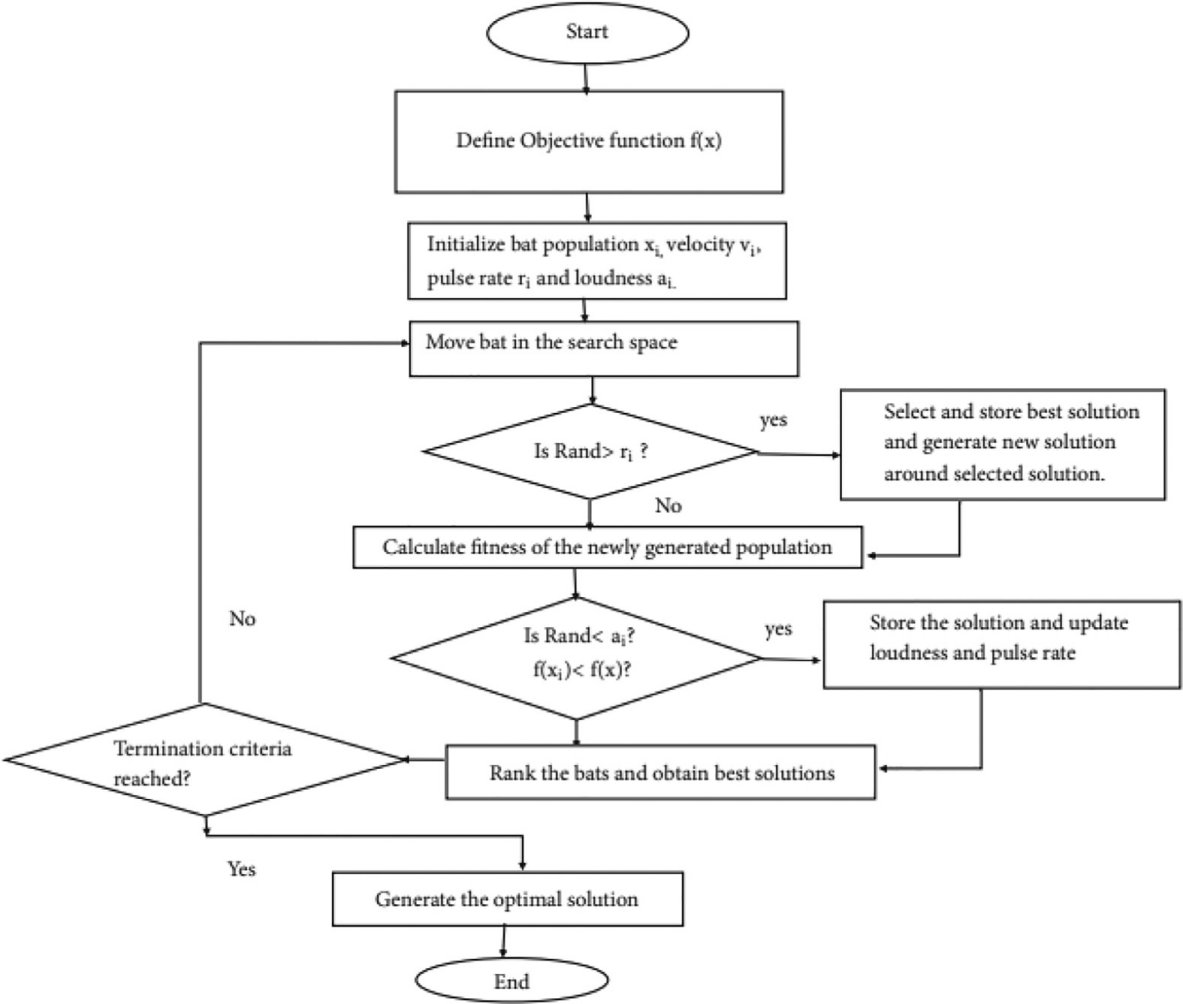
Flowchart of Bat Algorithm (BA). Fig. notes: In this flowchart, the operation of each step of the algorithm is presented in the form of a command box. This flowchart shows the execution steps of a BA.

The performance of BA algorithms is shown in the flowchart in Fig. 3.

## Improved bat algorithm (ISABA)

In this research, it is tried to improve the ISABA's performance by modifying the relationships related to the position and speed of each bat as an optimization solution. As mentioned, BA is a fast algorithm in finding answers, in this research, we try to correct Eq. (3) to increase the response speed of the algorithm. To this end, Eq. (7) is obtained as a modified position change relationship for each bat as an optimization solution.

(7) $$x_{i}^{t}=x_{*}+v_{i}^{t}$$

The change in the fly pattern of bats allows each bat to move to the optimal position. This change helps the algorithm to converge. The problem with this relationship is that the algorithm is trapped in local optima. For this purpose, a measure must be devised so that the algorithm examines the entire search space to find the main answer. In this study, the mutation operator works by adding a random constant in the search space range to the current position matrix of each bat and the optimal position. This operator examines the entire search space, making the algorithm highly capable of finding the optimal place for problems. The mutation operator is randomly applied to several population members and a certain proportion of the position matrix parameters in each solution.

As can be seen in Eq. (7), the position of each bat is a function of the optimal location and velocity.

Optimal Deviation: Due to the optimal deviation and since the speed of each bat reaches zero after hunting the prey, this idea cannot deviate from the optimal position of any solution. Therefore, the self-matching coefficient w can be used to zero the velocity vector when reaching the original optimum. So, Eq. (2) related to the velocity vector of each bat is modified as Eq. (8).

(8) $$v_{i}^{t}=w.\left(v_{i}^{t-1}+\left(x_{i}^{t-1}-x_{*}\right)f_{i}\right)$$

The coefficient w is optimized so that the position vector does not deviate when it reaches the location. This coefficient is adaptable and variable during the execution of the algorithm at each step.

The optimal side of this coefficient will tend to zero. The relation w is in the form of Eq. (9).

(9) $$w=x_{{*}_{i}}-x_{{*}_{i-1}}$$

where $x_{{*}_{i}}$is the vector of the current optimal position and $x_{{*}_{it}}$is the vector of the optimal position in the previous iteration.

## Investigating the dimensions of globalization

### Globalization

Globalization is a multifaceted phenomenon that has found its way into the various contexts of social, economic, political, cultural, military, technological, and environmental action. The International Monetary Fund defines globalization as the growing economic interdependence of countries around the world through the increase in the volume and variety of cross-border transactions in goods and services, as well as the international flow of capital and the wider dissemination of technology. The political dimension of globalization is to reduce the role of government and increase the role of nations and the authority of transnational corporations and organizations [20]. There are three processes in the globalization of the economy: transfer of goods, transfer of services, and transfer of capital. The globalization process will cause goods, services, and capital to quickly across the national borders and create changes within independent governments. The globalization of trade is the first manifestation of the globalization of the economy. In general, globalization includes economic, social, and political dimensions.

### Poverty and its dimensions

Although the phenomenon of poverty has different dimensions such as educational poverty, health, and income, in most studies, only the consumption aspect is considered with its income, because other aspects of poverty are directly related to it. In other words, they define poverty as a lack of income by consuming to meet basic needs. Amateur Kumar Sen pointed out that poverty should be considered as a deprivation of basic abilities and not just low incomes; this is the standard criterion for identifying poverty. Certainly, deprivation is a relative concept that may have different definitions in different places and times. What provides a good perspective for poverty analysis is that it expands our understanding of the nature and causes of poverty and deprivation. Booth & Rowntree in the late nineteenth century tried to distinguish between absolute and relative poverty. Absolute poverty is defined as the inability to achieve the minimum standard of and, thus, it depends on how the minimum wage is defined. Moreover, relative poverty is defined as the inability to achieve a certain standard of living that is deemed necessary or desirable in the current society. Hence, in the definition of relative poverty, the focus is on inequality in the distribution of income and wealth rather than the absolute amount of income of individuals. To achieve the depth of inequality in income distribution between individuals and households, the percentage of total income received by the poorest strata of society can be compared with the percentage of income received by the richest strata. It is of note that this concept is defined in different ways in different countries and has changed over time regarding economic developments [21].

### Economic globalization and poverty

Some economists are optimistic about the globalization of the economy because they value the positive performance of globalization and the economy. In their view, the more global the market is, the more efficient the allocation of resources will be. In line with this view, evidence shows that free-market economies have grown twice as fast as closed economies. In recent years, the stage of globalization has reached its peak and the number of people living in absolute poverty has decreased [22]. According to the pessimistic view, globalization is an unequal process with unequal distribution of profits and languages. This inequality leads to the polarization of rich and poor countries, on one hand, and rich and poor groups within countries, on the other [23].

### Social globalization and poverty

In the social dimension, globalization indicates the transformation of villages into cities and the transformation of cities into metropolises. Undoubtedly, globalization affects the environment. There are two main views between the proponents and the opponents. According to proponents, globalization leads to capital inflows and economic growth in developing countries. Increasing economic growth will counteract any improvement in the state of the environment. Poverty in many developing countries has led to degraded environmental quality, deforestation, and damage to natural resources. Also, communication and the Internet as one of the effects of social globalization has shrunk the gap between rich and poor countries. This trend is accelerating in recent decades, although its effect is not limited to the economy and in the social sphere and culture has been effective in this regard. Besides, the globalization process has caused many social and cultural problems to go beyond national borders and become global problems. Furthermore, globalization has brought about many social changes such as the greater vulnerability of poor and less developed societies to its crises and new cultural battles [24].

### Political globalization and poverty

Political globalization has led to political development in developing countries. Moreover, it has reduced the importance of geographical, political, and cultural borders and narrowed the scope of authority and power of national governments. In the field of political economy, the globalization trend has reduced the policy-making space of the government in the field of economic affairs such that governments have now less freedom to choose their economic policies. Thus, globalization not only increases poverty and underdevelopment, but is also a key solution to poverty reduction, political development, and cultural development. Also, the governments of industrialized countries subsidize their agricultural products for political reasons, making it difficult for developing countries to compete, while pressuring developing countries to subsidize their industrial products [25]. Furthermore, in the political realm, according to some experts, globalization has led to the emergence of invisible global decision-making centers and has threatened weak countries.

According to the theoretical foundations and previous studies, the model used in this study investigates the effect of economic, social, and political globalization on poverty in Iran (Eq. 10).

(10) HDI=f(KOFE;KOFS;KOFP;GDPG;FDI;IT;IUI;IN)

where the dependent variable is an indicator of human development, which was selected as an indicator to study poverty. KOFE shows the index of economic globalization. KOFS represents the index of social globalization. KOFP shows the index of political globalization. GDPG is GDP growth. FDI stands for foreign direct investment. IT represents international tourism. IUI stands for internet users and IN for Inflation. The data in this paper were obtained from the World Bank and the Federal Institute for Technology Economics at ETH (Eldgenossische Technische Hochschule Zurich) for 1995–2017 in Iran.

GDP represents only a part of macroeconomic activity. For this reason, in addition to GDP per capita, other important information such as socio-economic development and quality of life should be considered. One analytical tool that meets this goal is the HDI Human Development Index. This index is the average of the success achieved in a country in the three main dimensions of human development, namely:

(1) Long and healthy life (life expectancy at birth)
(2) Acquisition of knowledge (expected school years, average school years)
(3) Decent living standards (GDP per capita)

Measures

Human Development Index, which was introduced by the United Nations Human Development Program in 2000, was used to measure the well-being of individuals in a particular community. Also, it is used as one of the indicators to measure poverty. This index is graded between 0 and 1 [26]. In this article, this index was selected as a criterion for studying poverty in Iran. Attracting FDI without proper oversight and policy will lead to a polarization of wages in the economy by increasing workers’ wages in parts of production. It also diverts the production of luxury and expensive goods. This is possible given that low-income countries, such as Iran, have little control over the management of incoming FDI. Since developing countries have always faced the problem of capital shortages, the inflow of capital from developed countries increases the capital stock in developing countries. This increases labor productivity, income, and employment rate, as well as providing a large workforce in these countries. Thus, an increase in investment leads to a corresponding increase in poverty in developing countries. Increasing GDP in developing countries will increase per capita income and thus reduce poverty. Tourism creates many job opportunities and can reduce poverty. Rising inflation, on the other hand, severely hurts people with fixed nominal incomes, reduces their purchasing power, and benefits those with fixed asset reserves. Because the number of people with fixed incomes is usually higher than the number of capitalists and producers, rising inflation can lead to increased poverty in society. Another variable affecting poverty in communities is the amount of Internet access. People who do not have access to education due to long distances or the high cost of some facilities can easily increase their level of awareness through the Internet at the lowest cost. Such an approach would even create home-based businesses and entrepreneurship for people. An increase in the KOFE, as an index of economic globalization, can be due to an increase in exports, an increase in imports, or an increase in both variables. If the increase in this index is due to the increase in exports, employment will increase and reduce poverty. But if this increase in KOFE is due to an increase in imports, it will lead to an increase in inflation, followed by an increase in poverty. Thus, the globalization of the economy through poverty increases poverty and reduces poverty through unemployment. The KOFS index reflects social globalization. Communication services such as the Internet have increased public communication. Therefore, it can be stated that globalization in its social dimension can affect poverty in societies. The KOFP index indicates political globalization. Globalization has intensified the process of polarization of the world such that the gap between north and south is deepening and widening every day. On a global scale, the costs of globalization are also significant. The costs of globalization have outweighed its benefits because globalization has damaged the environment, corrupted political processes, slowed change, and prevented countries to adapt it while maintaining their cultures. Also, the crises that have led to widespread unemployment have resulted in long-term issues such as social collapse. Therefore, the political dimension of globalization is one of the variables affecting poverty in countries. Due to the complexity of the issues surrounding the impact of different dimensions of globalization on poverty, the ISABA method was chosen for modeling. In this paper, regression analysis (linear-power) was applied to investigate the impact of different dimensions of globalization on poverty in Iran. The models studied in this paper are developed in two linear-power forms. The linear type of the model is the effect of globalization on poverty according to Eq. (11):

(11) $$Y_{\mathrm{linear}}=b_{1}Z_{1}+b_{2}Z_{2}+b_{3}Z_{3}+b_{4}Z_{4}+b_{5}Z_{5}+b_{6}Z_{6}+b_{7}Z_{7}+b_{8}Z_{8}+b_{9}$$

According to economic theories, each of the coefficients of the linear form represents the extent to which the independent variable affects the dependent variable while other conditions are assumed to be constant. The power form of the model is the effect of globalization on poverty, according to Eq. (12):

(12) $$Y_{\mathrm{power}}=b_{1}{Z_{1}}^{b_{2}}*{Z_{2}}^{b_{3}}*{Z_{3}}^{b_{4}}*{Z_{4}}^{b_{5}}*{Z_{5}}^{b_{6}}*{Z_{6}}^{b_{7}}*{Z_{7}}^{b_{8}}*{Z_{8}}^{b_{9}}$$

In the power form, after taking the logarithms of the Eq. sides, each coefficient shows the relationship between the sensitivity or elasticity of the dependent variable and the independent variable. In Eqs. (11) and (12), Y denotes the dependent variable of the model and $Z_{i}$ shows the model's independent variables.

## Method optimization and validation

This paper aims to investigate the various dimensions of globalization on poverty in Iran using ISABA algorithms. The FITNESS function in this optimization is in the form of Eq. (13) [27]:

(13) $$\mathrm{Min}\;F\left(Z\right)=\sum_{i=1}^{k}{\left(y_{\mathrm{actual}}^{i}-y_{\mathrm{estimated}}^{i}\right)}^{2}$$

where $y_{\mathrm{actual}}^{i}$ represents the actual HDI values, $y_{estimated\;}^{i}$shows the values estimated for the HDI, and K indicates the number of observations. Also, MAE, MSE, and RMSE are error criteria used to compare the performance of linear-power models to provide the best data analysis. These errors are determined according to Eqs. (14–16).

(14) $$\mathrm{MSE}=\frac{1}{n}\sum_{i=1}^{23}{\left(y_{\mathrm{actual}}^{i}-y_{\mathrm{estimated}}^{i}\right)}^{2}\;\mathrm{for}\;n=1,2,\ldots ,23$$

(15) $$\mathrm{MAE}=\frac{1}{n}\sum_{i=1}^{23}\left|\;y_{\mathrm{actual}}^{i}-y_{\mathrm{estimated}}^{i}\right|\mathrm{for}\;n=1,2,\ldots ,23$$

(16) $$\text{RMSE}=\sqrt{\frac{1}{n}\sum_{i=1}^{23}{\left(y_{\mathrm{actual}}^{i}-y_{\mathrm{estimated}}^{i}\right)}^{2}\;\mathrm{for}\;n=1,2,\ldots ,23}$$

The designed network was trained based on data of 1995-2012 and evaluated using the data of 2013-2017 [27]. In this paper, the steps taken to model the effect of globalization on poverty are as follows:

Step 1: All input and output variables of the model were normalized according to Eq. (17).
  (17) $$Z_{N}=\left(Z_{R}-Z_{\min}\right)/\left(Z_{\max}-Z_{\min}\right)$$
where $Z_{N}$ denotes the normalized values of the variables and $Z_{R}$ represents the variables’ actual values. $Z_{\max}$ and $Z_{\min}$ specify the minimum and maximum values of each variable in the time series, respectively. Table 1 shows the minimum and maximum values of each of the research variables [28].

**Table 1:** Values for normalization.

	$Z_{\text{max}}$	
Economic Globalization Index	44.845	16.279
Social Globalization Index	69.542	25.867
political globalization Index	81.124	52.409
GDP growth (annual %)	13.396	-7.445
Foreign direct investment, net inflows (BoP, current US$)	5.02E+09	1.7E+07
International tourism, receipts (current US$)	4.77E+09	1.42E+08
Individuals using the Internet (% of the population)	64.044	0.004
Inflation, consumer prices (annual %)	49.656	8.651
Human Development Index	0.799	0.640

Table notes: This table shows the minimum and maximum values of each of the article variables. These values are used to normalize the data used in this article.
Step 2: According to the FITNESS function defined for BA algorithms and ISABA algorithms, the algorithms are trained using the data from 1995-2012. Next, the coefficients of each linear-power model were obtained. Table 2 shows the factors of each research model [28,29].

**Table 2:** The best-obtained coefficient by BA algorithms and ISABA algorithms.

	B1	B2	B3	B4	B5	B6	B7	B8	B9
**Y_BA-Linear_**	0.043	-0.080	0.470	0.121	0.082	-0.124	-0.229	0.137	0.090
**Y_BA-power_**	-0.244	0.264	-0.288	0.105	0.063	0.030	0.141	-0.011	0.511
**Y_ISABA-Linear_**	-0.119	0.312	0.305	0.215	-0.529	0.643	-0.225	-0.065	-0.024
**Y_ISABA-power_**	-0.016	-0.740	0.818	0.871	0.627	-0.894	-0.172	0.106	0.512

Table notes: This table presents the coefficients of each research model. According to the functions introduced for estimation, the corresponding coefficients are obtained from the result of the implementation of the ISABA algorithm and BA algorithm.
Step 3: Up to this point, the algorithms have been trained using 1995-2012 data. At this stage, to measure the performance of the algorithm, the data of 2013-2017 were introduced to the algorithm as test data and were evaluated by comparing the output of the algorithm and the actual values of the performance of the algorithms. The evaluation criterion in this section is relative error. The relative error percentage of BAs for test data is shown in Table 3. The corresponding error percentage of trained BA algorithms for test data is presented in Table 4. Table 5 also indicates the MAE, MSE, and RMSE error criteria for all 1995-2017 data.

**Table 3:** Comparison of the BA models HDI in the test period (2013-2017).

	HDI	Y_BA-Linear_	Relative error (%)	Y_BA-power_	Relative error (%)
**2013**	0.785	0.720	-0.081	0.707	-0.099
**2014**	0.788	0.696	-0.116	0.718	-0.087
**2015**	0.789	0.688	-0.127	0.714	-0.094
**2016**	0.799	0.701	-0.121	0.799	0.000
**2017**	0.799	0.692	-0.133	0.727	-0.089
**Average**	-	-	-0.116	-	-0.074

Table notes: This table presents the test results of the BA for the research functions. The values in this table show the relative estimation error value for the test data so that the accuracy of the estimate can be assessed.

**Table 4:** Comparison of the ISABA models HDI in the testing period (2013-2017).

	HDI	Y_ISABA-Linear_	Relative error (%)	Y_ISABA-power_	Relative error (%)
**2013**	0.785	0.720	0.001	0.707	-0.140
**2014**	0.788	0.696	0.013	0.718	-0.114
**2015**	0.789	0.688	9.75E-05	0.714	-0.065
**2016**	0.799	0.701	-0.017	0.799	0.000
**2017**	0.799	0.692	-0.016	0.727	0.158
**Average**	-	-	-0.003	-	-0.032

Table notes: This table presents the test results of the ISABA algorithm for the research functions. The values in this table show the relative estimation error value for the test data so that the accuracy of the estimate can be assessed.

**Table 5:** Comparison of performance evaluation of BA algorithms and ISABA algorithms in linear and power models.

Error Histogram			
Algorithm Model	MSE	MAE	RMSE
Y_BA-Linear_	0.003	0.041	0.051
Y_BA-power_	0.002	0.034	0.050
Y_ISABA-Linear_	0.000	0.012	0.016
Y_ISABA-power_	0.004	0.052	0.063

Table notes: This table shows the values of the model measurement criteria. By comparing these values, we can observe the accuracy of each of the functions and algorithms and introduce the best model and the most accurate algorithm for modeling.

**Fig. 4:**
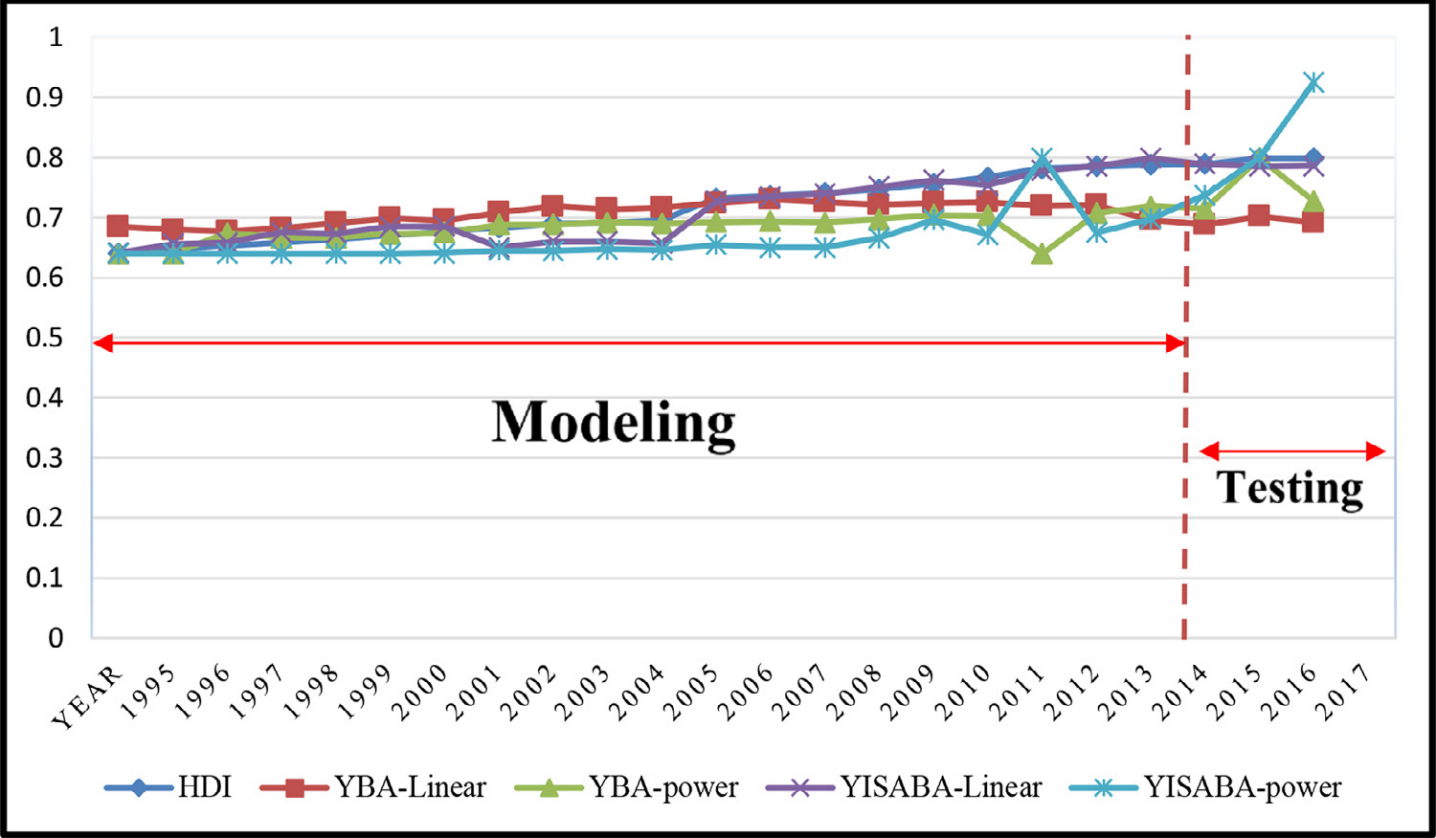
Performance of BA algorithms and ISABA algorithms for HDI models. Fig. notes: This diagram shows the modeling results of each of the functions and algorithms. By comparing each of the graphs with the actual value of the human development index, the most accurate model for modeling the problem can be selected.

To observe the performance of ISABA algorithms compared to BA algorithms, the output of research models is shown in Fig. 4.

## Conclusion

This research aims to evaluate the performance of the improved bat algorithm (BA) compared to the BA. For this purpose, the study of the impact of different dimensions of globalization on poverty in Iran was used as a non-classical structure. The results of this article showed that the linear model of the article is more appropriate than other algorithms to explain the impact of globalization on poverty. Also, the existence of minimum error criteria in the BA compared to the BA indicates the proper performance of this algorithm in converging to the response. ISABA in the linear model of the article with MSE of 0.00025 and MAE of 0.01173 was able to provide a suitable model for poverty in Iran with an average relative error of 0.3%. Based on the results of the linear model using the ISABA method, it can be stated that the economic dimension of globalization has reduced poverty in Iran because the increase in exports has led to a decrease in unemployment and poverty in this country. Nevertheless, the social and political dimension of globalization has increased poverty in Iran. Also, the growth of GDP in Iran has increased poverty, which can be attributed to the disproportion among manufacturing and inadequate distribution industries. Furthermore, proper management in FDI absorption has led to an increase in FDI, leading to poverty reduction in Iran. Another noteworthy issue is that tourism development in Iran has increased poverty. Finally, the widespread use of the Internet and the platform it provides for business and entrepreneurship development has reduced poverty in Iran.

## Declaration of Competing Interest

The authors declare that they have no known competing financial interests or personal relationships that could have appeared to influence the work reported in this paper.

## Appendix Supplementary materials

Image, application 1
